# Non-clostridial gas gangrene due to Anaerococcus tetradius following a cesarean section: A case report

**DOI:** 10.1016/j.bjid.2025.104609

**Published:** 2025-12-23

**Authors:** Takehiro Hashimoto, Mamiko Okamoto, Tomonori Yamada, Eiji Kobayashi, Kazufumi Hiramatsu

**Affiliations:** a Hospital Infection Control Center, Oita University Hospital, Oita, Japan; b Department of Obstetrics and Gynecology, Faculty of Medicine, Oita University, Oita, Japan

**Keywords:** Non-clostridial gas gangrene, *Anaerococcus tetradius*, Cesarean section

## Abstract

A 25-year-old healthy woman was admitted to our hospital with fever and abdominal pain. Abdominal contrast-enhanced computed tomography revealed a fluid collection with foamy gas formation extending from both rectus abdominis muscles to the subcutaneous tissue. Antimicrobial therapy with meropenem (3 g/day), clindamycin (1800 mg/day), and daptomycin (300 mg/day) was initiated as empiric therapy. Subcutaneous abscess culture revealed gram-positive cocci, which was identified as *Anaerococcus tetradius*. Histopathological examination of the necrotic tissue showed skeletal muscle necrosis. On the basis of these findings, the patient was diagnosed with Non-Clostridial Gas Gangrene (NCGG) due to *A. tetradius*. We report a novel case of NCGG caused by *A. tetradius* following a cesarean section in an immunocompetent patient. Although *A. tetradius* has been detected in clinical specimens, cases of human infections are extremely rare. Clinicians should consider *A. tetradius* infection as a complication of a cesarean section.

## Introduction

Gas gangrene is a rapidly progressive, life-threatening infection characterized by the abrupt onset of severe pain, high fever, extensive local swelling, widespread myonecrosis, and gas formation at the infection site^1^. Non-Clostridial Gas Gangrene (NCGG), although rare, represents another form of necrotizing infection and is associated with a high mortality rate. It often developing in patients with underlying conditions such as diabetes mellitus^2^. *Anaerococcus tetradius* is a strictly anaerobic, gram-positive, coccoid that forms tetrads and is part of the normal vaginal flora^3^. Although *A. tetradius* has been detected in clinical specimens, only one case of human infection, a case of bacteremia with unknown clinical details, has been reported^4^. Hitherto, no detailed reports of *A. tetradius* infection in humans have been published, nor has NCGG been documented following a cesarean section in immunocompetent individuals. Here, we report a case of NCGG caused by *A. tetradius* following a cesarean section in an immunocompetent patient.

## Case presentation

**Fig. 1:**
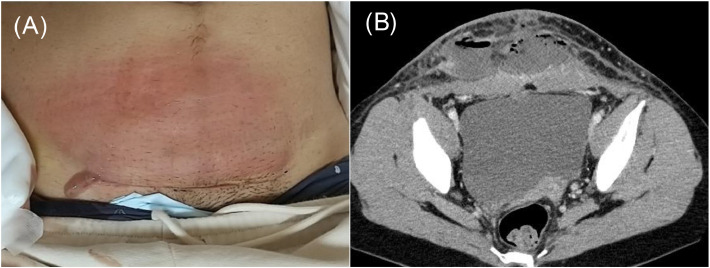
(A) Abdominal findings: the erythema, warmth, and tenderness noted in a band around the head of the wound in the lower abdomen and pus discharge from the right end of the wound. (B) Contrast-enhanced computed tomography scan revealing a fluid collection with foamy gas formation extending from both rectus abdominis muscles to the subcutaneous tissue. The lesion was encapsulated by an enhancing rim and surrounded by fat strands, suggestive of abscess formation.

**Fig. 2:**
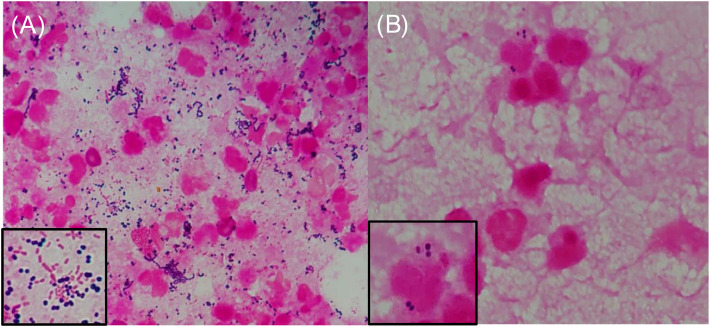
(A) Gram staining of the wound swab revealing White Blood Cell (WBC) 3+, Gram-Positive Cocci (GPC) 3+, and gram-negative cocci 3+. (B) Gram staining of the subcutaneous abscess showing WBC 3+ and GPC+.

A healthy 25-year-old woman underwent a cesarean section at 38-weeks and 2-days of gestation because of cephalopelvic disproportion. Clindamycin and panimycin were administered for perioperative antibiotic prophylaxis. She was discharged on postoperative day-7, but developed a fever of 38 °C and incisional pain the following day. Despite receiving clarithromycin, her condition worsened, with her fever rising to 39.6 °C and increasing pain at the incision site, prompting a referral to our hospital. At admission, she had a fever of 38.9 °C, and erythema, warmth, and tenderness were noted in a band around the wound head in the lower abdomen, in addition to pus discharge from the right end of the wound (Fig. 1A). Laboratory test results on admission showed a markedly increased White Blood Cell (WBC) count (28,410 μL) and C-reactive protein level (24.5 mg/dL). Contrast-enhanced computed tomography revealed a fluid collection with foamy gas formation extending from both rectus abdominis muscles to the subcutaneous tissue. The lesion was encapsulated by an enhancing rim and surrounded by fat strands (Fig. 1B). Meropenem (3 g/day), clindamycin (1800 mg/day), and daptomycin (300 mg/day) were initiated after obtaining two sets of blood cultures, wound swab culture, and subcutaneous abscess culture. Gram staining of the wound swab revealed WBC 3+, Gram-Positive Cocci (GPC) 3+, and gram-negative cocci 3+ (Fig. 2A), whereas staining of the subcutaneous abscess showed WBC 3+ and GPC + (Fig. 2B). On day-2, surgical debridement of the necrotic tissue around the fascia and wound irrigation were performed for infection control. On day-5, blood cultures were negative; *Enterococcus faecalis, Peptoniphilus harei*, and *A. tetradius* were isolated from wound swabs. *A. tetradius*, which was identified using matrix-assisted laser desorption/ionization time-of-flight mass spectrometry (Bruker Daltonics, GmbH, Bremen, Germany; score value, 2.063), was also isolated from the subcutaneous abscess. The minimum inhibitory concentrations for the organism, determined using the broth microdilution method, were as follows: amoxicillin, 1 µg/mL; piperacillin, ≤ 1 µg/mL; ampicillin/sulbactam, ≤ 0.25 µg/mL; piperacillin/tazobactam, ≤ 1 µg/mL; cefmetazole, ≤ 0.5 µg/mL; cefotaxime, ≤ 0.5 µg/mL; cefepime, ≤ 0.5 µg/mL; meropenem, ≤ 0.5 µg/mL; clarithromycin, 0.25 µg/mL; clindamycin, ≤ 0.12 µg/mL; and metronidazole, ≤ 0.5 µg/mL. Clindamycin was discontinued on day-6 because no toxin-producing bacteria were detected. On day-9, histopathological examination of the necrotic tissue revealed dense neutrophilic infiltration in the adipose tissue, fibrous stroma, and skeletal muscle; hemorrhage; and congestion. Some skeletal muscle areas showed necrosis. On the basis of these findings, the patient was diagnosed with NCGG caused by *A. tetradius*. Consequently, we recommended discontinuing daptomycin on the basis of the established diagnosis. However, combination therapy with meropenem and daptomycin was continued because the attending physicians remained concerned about the possibility of complications caused by other infections. Meropenem and daptomycin were discontinued on day-16. Wound closure was performed on day-23, and the patient was discharged on day-31. She remained clinically stable without gas gangrene recurrence for 1-year after discharge. Because *A. tetradius* infection in humans is rare, 16S ribosomal RNA gene sequencing was performed on the isolates. Sequence analysis using the BLAST homology search tool (https://blast.ncbi.nlm.nih.gov/Blast.cgi) revealed a 98.8 % identity with *A. tetradius* JCM 1964 (GenBank accession n° LC036320.1), with 1446 out of 1463 bp nucleotide matches.

## Discussion

Infectious gangrene is a rapidly progressive form of cellulitis characterized by extensive necrosis of the subcutaneous tissue, overlying epidermis, and dermis. It is classified into six main types based on causative organisms, infection site, and predisposing factors. NCGG falls under the category of Synergistic Necrotizing Cellulitis (SNC). SNC is a subtype of necrotizing fasciitis characterized by severe necrotic lesions resulting from mixed infections with aerobic and anaerobic bacteria. The incubation period is 3–14 days, and the disease typically presents acutely, with prominent localized pain and tenderness. Gas formation within the tissue is observed in approximately 25 % of cases, and bacteremia develops in approximately 50 %. However, necrotizing fasciitis typically presents after an incubation period of 1–4 days and progresses rapidly with acute onset of severe pain, marked swelling, and erythematous cellulitis accompanied by patches of skin necrosis. Purulent exudate, gas formation, and a foul odor are frequently observed. Systemic toxicity is usually pronounced^5^^,^^6^. Unlike necrotizing fasciitis, NCGG affects the fascia and the underlying muscles. In our case, histopathological examination of the necrotic tissue showed skeletal muscle necrosis, and the patient was diagnosed with NCGG. The causative organisms are typically a combination of facultative anaerobes (e.g., *Escherichia coli, Klebsiella* species, and various streptococci) and obligate anaerobes (e.g., *Bacteroides* and *Peptostreptococcus* species)^5^^,^^6^. According to reports from Japan, the most commonly isolated aerobic organisms include streptococci, *E. coli*, enterococci, *Staphylococcus aureus, Klebsiella* species, and *Proteus* species. Among anaerobes, *Bacteroides* and *Peptostreptococcus* species have been frequently identified^7^. More than 90 % of NCGG cases are caused by polymicrobial infections, with there being only a few documented cases in which NCGG was attributed to a single bacterium^8^^,^^9^. In our case, *Enterococcus faecalis* 2+, *Peptoniphilus harei* 3+, and *A. tetradius* 3+ were isolated from wound swabs. We assessed semi-qualitative wound cultures using a semi-quantitative grading scale for bacterial burden: ± (scanty), 1+ (few), 2+ (moderate), and 3+ (numerous)^10^. However, superficial skin swabs are inherently low-quality specimens and offer limited clinical value due to their low sensitivity and specificity^11^. In Fournier gangrene, the concordance between deep tissue cultures and superficial swabs has been reported to be only 27.8 %^12^. Therefore, we determined the infection to be monomicrobial based on the Gram stain findings and culture results. Nevertheless, clinicians should be aware that anaerobic wound infections often include mixed flora. To our knowledge, there are no previous reports of NCGG caused by *A. tetradius. A. tetradius* was previously known as *Peptostreptococcus tetradius* before being reclassified into the genus *Anaerococcus*^13^. Although *A. tetradius* has been detected in clinical specimens^3^, no detailed reports on *A. tetradius* infection in humans have been published^4^. Additionally, NCGG is exceedingly rare in immunocompetent individuals. Reported cases have been limited to post-traumatic settings, such as roadside injuries^2^, and hitherto, no cases have been documented following cesarean section in immunocompetent patients.

A few cases of aggressive soft-tissue infection have been reported after a cesarean section^14^, ^15^, ^16^, ^17^. The risk factors for aggressive soft-tissue infection include diabetes mellitus, hypertension, cirrhosis, obesity, malignancy, chronic disease, malnutrition, advanced age (>50-years), and use of nonsteroidal anti-inflammatory drugs in the early postoperative period^17^. There were no such risk factors identified in our case. Additionally, *Streptococcus pyogenes* produces multiple proteases and virulence factors that promote rapid tissue destruction in aggressive soft-tissue infections^18^. In contrast, severe soft-tissue infections caused by commensal vaginal anaerobes are rarely documented, and their molecular and clinicopathological characteristics remain poorly understood^3^. Thus, further research is required to clarify the mechanism of aggressive soft-tissue infections caused by commensal vaginal anaerobes.

Because clindamycin, penicillin, and moxifloxacin resistance has been reported in *Anaerococcus* species, clinicians consider amoxicillin/clavulanate, metronidazole, imipenem/cilastatin, and piperacillin/tazobactam as initial treatment options^4^. However, data on the antimicrobial susceptibility of *A. tetradius* are limited. Because no breakpoints have been established for *A. tetradius*, antimicrobial susceptibility was assessed using the Clinical and Laboratory Standards Institute guidelines^19^. Our results indicate that the isolate from our patient was susceptible to most antimicrobial agents, except amoxicillin.

## Conclusion

We reported a case of NCGG caused by *A. tetradius* following a cesarean section in an immunocompetent patient. Currently, there are no established standard antimicrobial treatments for *A. tetradius* infection. Although the *A. tetradius* detection rate in clinical settings remains extremely low, the organism may be under-recognized. Further research is required to clarify the epidemiological and clinical characteristics of *A. tetradius* infections.

## Consent for publication

Informed consent was obtained from the patient for publication of this case report and accompanying images.

## Funding

This research did not receive any specific grant from funding agencies in the public, commercial, or not-for-profit sectors.

## Data availability statement

The data that support the findings of this study are available from the corresponding author upon reasonable request.

## Conflicts of interest

The authors declare no conflicts of interest.
